# Students in a Course-Based Undergraduate Research Experience Course Discovered Dramatic Changes in the Bacterial Community Composition Between Summer and Winter Lake Samples

**DOI:** 10.3389/fmicb.2021.579325

**Published:** 2021-02-18

**Authors:** Stokes S. Baker, Mohamed S. Alhassan, Kristian Z. Asenov, Joyce J. Choi, Griffin E. Craig, Zayn A. Dastidar, Saleh J. Karim, Erin E. Sheardy, Salameh Z. Sloulin, Nitish Aggarwal, Zahraa M. Al-Habib, Valentina Camaj, Dennis D. Cleminte, Mira H. Hamady, Mike Jaafar, Marcel L. Jones, Zayan M. Khan, Evileen S. Khoshaba, Rita Khoshaba, Sarah S. Ko, Abdulmalik T. Mashrah, Pujan A. Patel, Rabeeh Rajab, Sahil Tandon

**Affiliations:** ^1^Biology Department, University of Detroit Mercy, Detroit, MI, United States; ^2^School of Environment and Sustainability, University of Michigan, Ann Arbor, MI, United States; ^3^Mike Ilitch School of Business, Wayne State University, Detroit, MI, United States

**Keywords:** 16S rRNA, aquatic, Course-based Undergraduate Research Experience, eutrophic, ice, Laboratory Course Assessment Survey, metabarcoding, metagenomics

## Abstract

Course-based undergraduate research experience (CURE) courses incorporate high-impact pedagogies that have been shown to increase undergraduate retention among underrepresented minorities and women. As part of the Building Infrastructure Leading to Diversity program at the University of Detroit Mercy, a CURE metagenomics course was established in the winter of 2019. Students investigated the bacterial community composition in a eutrophic cove in Lake Saint Clair (Harrison Township, MI, United States) from water samples taken in the summer and winter. The students created 16S rRNA libraries that were sequenced using next-generation sequencing technology. They used a public web-based supercomputing resource to process their raw sequencing data and web-based tools to perform advanced statistical analysis. The students discovered that the most common operational taxonomic unit, representing 31% of the prokaryotic sequences in both summer and winter samples, corresponded to an organism that belongs to a previously unidentified phylum. This result showed the students the power of metagenomics because the approach was able to detect unclassified organisms. Principal Coordinates Analysis of Bray–Curtis dissimilarity index data showed that the winter community was distinct from the summer community [Analysis of Similarities (ANOSIM) *r* = 0.59829, *n* = 18, and *p* < 0.001]. Dendrograms based on hierarchically clustered Pearson correlation coefficients of phyla were divided into a winter clade and a summer clade. The conclusion is that the winter bacterial population was fundamentally different from the summer population, even though the samples were taken from the same locations in a protected cove. Because of the small class sizes, qualitative as well as statistical methods were used to evaluate the course’s impact on student attitudes. Results from the Laboratory Course Assessment Survey showed that most of the respondents felt they were contributing to scientific knowledge and the course fostered student collaboration. The majority of respondents agreed or strongly agreed that the course incorporated iteration aspects of scientific investigations, such as repeating procedures to fix problems. In summary, the metagenomics CURE course was able to add to scientific knowledge and allowed students to participate in authentic research.

## Introduction

For over a quarter of a century, reports from science, technology, engineering and mathematics (STEM) advisory organizations have been calling for reform of undergraduate STEM curricula to focus on developing analytical skills instead ofmemorizing content (Project Kaleidoscope, 1991; Howard Hughes Medical Institute, 1996; National Research Council, 1996, 2003; National Science Foundation, 1996; Bauerle et al., 2009). These same reports have called for teaching innovations that will increase the participation of underrepresented minority students in STEM. Programs that have met this goal have some of the following attributes: experience with authentic research, active learning, collaborative learning communities where students share an intellectual experience, and involvement in research that directly impacts their communities (Graham et al., 2013; Toven-Lindsey et al., 2015; Estrada et al., 2016; Provost, 2016). Faculty-supervised undergraduate research is a well-established approach to provide these high-impact activities. Unfortunately, the approach has limited capacity (i.e., only a few students can be effectively taught using an apprentice model). One strategy to overcome the bottleneck is to provide course-based undergraduate research experience (CURE) instruction (Provost, 2016; Bell et al., 2017).

Course-based undergraduate research experiences are defined as laboratory courses that incorporate the following attributes (Auchincloss et al., 2014; Provost, 2016):

1. Scientific Process: Conducting research as practiced by professional scientists.
2. Discovery: Investigating novel questions.
3. Relevance: Having impacts beyond the classroom because the research advances scientific knowledge.
4. Collaboration: Collectively tackling difficult problems.
5. Iteration: Conducting research built upon existing knowledge, learning by failure and retrying, and revising thinking after self-analysis and peer-critique.

Several CURE courses have been successfully implemented that involved microbiology, virology, molecular biology, bioinformatics, and other life science disciplines (Wang, 2017), including metagenomics (CUREnet, 2013; Lentz et al., 2017; Wang, 2017). One strength of CUREs is they can support distributive approaches to address large biological questions (Hatfull, 2015; Wang, 2017). Because the microbial world is so diverse and vast, the National Research Council has called for the incorporation of metagenomics into undergraduate biology instruction because it can be an effective distributive strategy to advance scientific knowledge (Jurkowski et al., 2007). An example of a successful distributive-science CURE is the Science Education Alliance Phage Hunters Advancing Genomics and Evolutionary Science (SEA-PHAGES) program (Hatfull, 2015).

The University of Detroit Mercy’s ReBUILD Detroit program (Snyder and Kumar, 2019) is part of a National Institutes of Health initiative to increase the pipeline of underrepresented minority undergraduates entering biomedical STEM research careers (National Institutes of Health, 2019). To recruit and retain the target population, ReBUILD Detroit is using a “persistence model” (Graham et al., 2013; Toven-Lindsey et al., 2015) which involves having the students participate in research activities every semester, including the first semester of their freshman year. To increase the availability of authentic research experiences for undergraduates and to support ReBUILD Detroit’s retention strategy, a CURE course entitled, “Applied Metagenomics” was established in the winter of 2019 and repeated in the winter of 2020. The course investigations focused on aquatic microbiology because water quality issues are important community concerns in metropolitan Detroit (Bolsenga and Herdendorf, 1993). Because Detroit is a large industrial city within the Great Lakes Basin, the students have a myriad of water quality issues they can investigate.

### Background Related to the Environmental Question Investigated by the Students

Metagenomics, as defined by the National Research Council (2007) and Wooley et al. (2010), is the study of uncultured microorganisms found in environmental samples, by use of massively parallel sequencing. The environmental DNA (eDNA) sequences can be bulk DNA (a.k.a., shotgun metagenomics) or amplicons from specific loci (a.k.a., metabarcoding). Metagenomic studies have shown that freshwater ecosystems appear to have a distinct assemblage of prokaryotes in the epilimnia. Metanalysis studies of 16S rRNA gene sequences obtained from diverse lakes (e.g., oligotrophic to highly eutrophic) on different continents have shown that freshwater lakes have an assemblage of prokaryotes that are distinct from marine and terrestrial habitats (Zwart et al., 2002; Newton et al., 2011).

Some 16S rRNA metabarcoding studies have shown that freshwater trophic status can impact the composition of prokaryote communities. For example, a study of human-impacted tributaries of the Great Lakes showed greater species richness in oligotrophic lake samples (Newton and McLellan, 2015). A similar pattern was observed in a separate study of the Great Lakes, canals, and streams of the Niagara Peninsula (Mohiuddin et al., 2019). In contrast, a study of oligotrophic versus eutrophic lakes in Greece showed greater species diversity in the eutrophic samples (Karayanni et al., 2019). These results suggest that trophic status can alter the freshwater prokaryote diversity, but a general rule on the relationship between nutrient level and prokaryote community diversity has not been established.

Many metagenomic investigations of aquatic ecosystems only sample water during ice-free months (for examples, see Shade et al., 2007; Mohiuddin et al., 2019). As a result, less information on the nature of aquatic bacterial communities in seasonally freezing lakes is available in the literature. Vigneron et al. (2019) observed that ice-covered tundra lakes had a rich prokaryotic community with similar cell densities to the ice-free water. However, the composition of the prokaryotic community changed with the seasons. Metabolic pathways deduced from shotgun metagenomic sequencing showed the prokaryotic community shifted from phototrophic and aerobic metabolism in the summer to reductive metabolism that could degrade aromatic organics in the winter. Tran et al. (2018) observed similar results in their investigations of Verrucomicrobia communities of taiga lakes. These results suggest that winter prokaryotic communities in ice-covered lakes contain a rich biota distinct from their open water counterparts. With these observations in mind, the goal of the students in Applied Metagenomics was to determine if the prokaryotic community in an ice-covered versus open-water temperate lake exhibited changes in community composition similar to those observed in tundra and boreal lakes.

## Materials and Methods

### Human Subject Statement

This study was carried out in accordance with the recommendations of National Institutes of Health’s Human Subjects Research Guidelines. The protocol was approved by the University of Detroit Mercy’s Institutional Review Board (Protocol Number 1718-53) on March 10, 2018. All subjects gave written informed consent in accordance with the Declaration of Helsinki.

### Class Description and Assessment

Applied Metagenomics (BIO3201) was offered at the University of Detroit Mercy during the winter terms of 2019 and 2020. The prerequisite for the course was genetics, cell and molecular biology, or biochemistry. In 2019, eight students were enrolled in the 15 week course. Their self-reported demographics were as follows: Gender: 75% males, 25% female; Ethnicity/Race: 75% white, 25% Asian/Pacific Islander. In 2020, 16 students took the course. Their self-reported demographics were as follows: Gender: 50% males, 43.75% female, 6.25% prefer not to answer. Ethnicity/Race: 37.5% White (Middle Eastern descent), 25% White (European descent), 31.25% Asian/Pacific Islander, 6.25% Black African American, and 6.25% prefer not to disclose. The sum is greater than 100% because some students reported themselves in more than one category. The course was taught twice weekly in 2 to 3 h sessions. During the first 2 weeks of the course, students performed skills-building activities involving accurate micro-pipetting, sterile technique, and basic bacteriology (i.e., pouring Petri plates, streak plates, and liquid transfers). After completing the skills-building portion of the course, the students conducted their investigations. Students’ grades were based on written laboratory reports and exams. In 2019, students elected to conduct a study to compare the prokaryote composition of summer versus winter aquatic communities. In 2020, students chose to study the prokaryotic community of two park ponds. In both terms, the students performed dilution plate count assays, field-collected water samples, and isolated eDNA. Due to the COVID-19 epidemic, the students in 2020 were unable to complete their study because the course was switched to an online format during the last 5 weeks. For the online component, the students independently analyzed the data generated by the 2019 students. Both years, students were taught how to interpret species accumulation curves (Knell, 2018), principal component analysis (Starmer, 2015, 2017), and hierarchically clustered heatmaps (Starmer, 2016) by watching online videos. In 2020, the instructor created a video tutorial on how to use MicrobiomeAnalyst (Dhariwal et al., 2017; Chong et al., 2020), which was posted on a course-management website.

To determine if the course provided the expected outcome of a CURE, the Laboratory Course Assessment Survey (LCAS) was administered during the last week of the course (Corwin et al., 2015). The LCAS is a validated psychometric instrument that assesses students’ views of the frequency of collaboration, perception of creating new scientific knowledge, and frequency they needed to repeat and evaluate their experimental results. To assess student attitudes regarding next-generation sequencing technologies, the Genome Consortium for Active Teaching – Sequencing Group (GCAT-SEEK) questionnaire (Buonaccorsi et al., 2011; Tobin and Shade, 2018) was administered the first week of the course and the last week of the course. Additionally, an end-of-term survey written by the instructor was given to the students as a qualitative assessment. All the surveys and questionnaires were taken anonymously.

### Study Site

**FIGURE 1:**
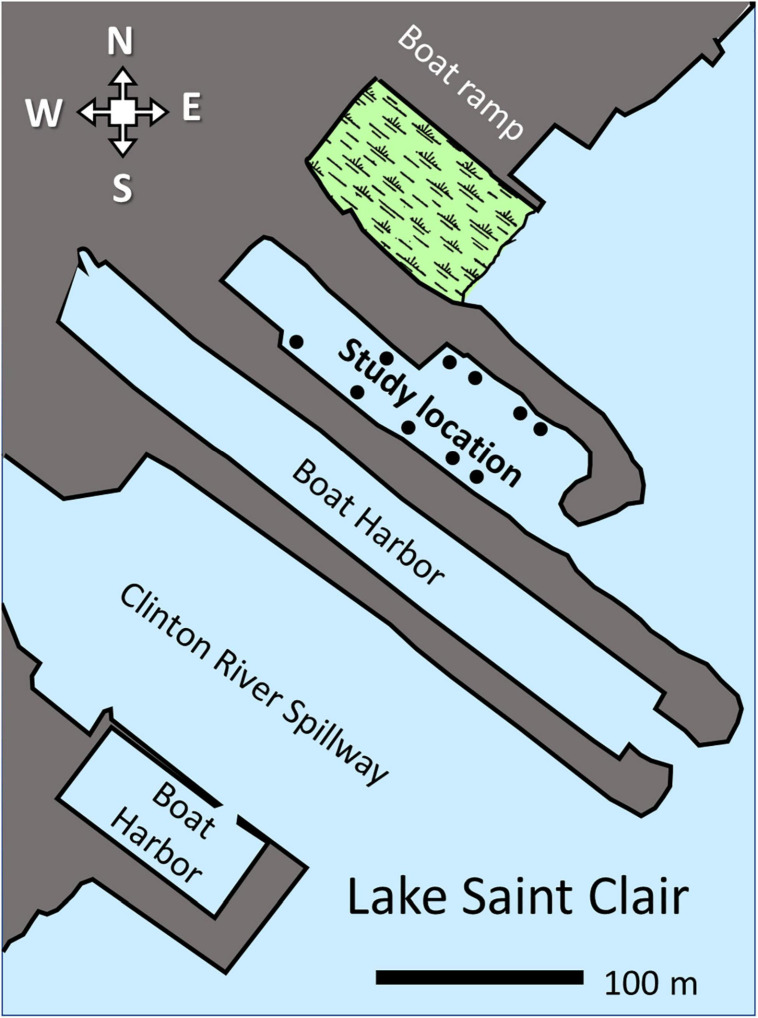
Map of the study’s location. Dots represent water sampling locations. Wetland contiguous to the study site is shaded in green. The range bar is 100 m.

Samples were taken from an artificial cove in Lake Saint Clair (Harrison Township, MI, United States; latitude 42.561496, longitude −82.843249; Figure 1). The cove was created when a stone and earth breakwater was installed to create a boat harbor. The cove is located next to the mouth of the Clinton River Bypass, a flood-control canal that can carry Clinton River sediments (Francis and Haas, 2006; Healy et al., 2008). The harbor was abandoned when the property was acquired by the Michigan Department of Natural Resources. Natural successional processes have been allowed to occur in the cove for several years. Sediments from the Clinton River Bypass have been accumulating. As a result, the water depth was approximately 1 to 2.5 meters, with the shallowest portion near the mouth of the harbor. A rich community of aquatic vegetation, invertebrates, fish, and turtles resided in the cove.

### Water Sampling

Water samples were collected in gamma-irradiated sterile bottles placed on ice and transported back to the laboratory. Surface water samples were collected in summer (June 22, 2016) from ten different locations (Figure 1). To exclude floating plant material, the water was filtered through autoclaved rayon polyester mesh (22–25 μm pore size) during collection. After collection, the bottles were capped with an airtight closure. The water temperature was 23°C. To collect water in the winter (February 5, 2019), an autoclaved ice auger (15 cm diameter) was used to drill holes through 10 cm to 61 cm of ice. The auger was sterilized with 95% ethanol between samplings. The holes were drilled near the same location as the ten summer water samples. The water temperature underneath the ice was 0.8°C. A surface sterilized pole was used to lower the collection bottle below the ice. An ethanol sterilized rubber stopper was removed from the mouth of the sampling bottles by pulling an attached string. Once recovered, the bottles were closed with an airtight cap. The samples were stored on ice until DNA extraction.

### Water and Sediment Analysis

After microfiltration (see section “DNA Extraction”), the sterile cove-water samples were placed into a −20°C freezer until analysis. On the day of analysis, the water samples were thawed in a room temperature water bath. Orthophosphate concentrations were measured using Hanna Instruments (Woonsocket, RI, United States) Ultra Low Range Phosphate Reagent kit, which is based on the ammonium molybdate-ascorbic acid method (Environmental Protection Agency, 1978). For orthophosphate analysis, 10 mL of water was transferred to virgin sterile polypropylene tubes. The content of the reagent packet was dissolved into the samples. After a 3 min room temperature incubation, the samples were transferred to a 5 cm long cuvette. The absorbance at 708 nm was measured. Winter samples were measured by Applied Metagenomics students. Summer samples were measured by students enrolled in Ecology Laboratory during the fall of 2019. Water hardness, ammonia, and nitrate levels were measured using Hanna Instruments model HI83200 Multiparameter Photometer kits.

During the fall 2018 semester, students enrolled in Ecology Laboratory performed chemical assays on the cove’s benthic sediments. Samples were collected by attaching a plastic beaker to a 3 m pipe. To remove the excess water from the sample, small colanders were lined with coffee filter paper and allowed to drip. The LaMotte (Chestertown, MD, United States) Soil Analysis Kit (5010-01) was used to measure phosphorous, potassium, nitrogen, and pH.

### DNA Extraction

Within 2 h of sampling, bacteria were isolated by passing the samples through gamma-irradiated disposable microfiltration (pore size 0.2 μm, diameter 47 mm) apparatuses. The apparatuses had closures to prevent contamination. Immediately after vacuum filtration, the apparatuses were moved to a laminar flow hood. Membranes were cut out using sterile scalpels, transferred to gamma-irradiated polystyrene Petri plates, and cut into small fragments. To prevent cross contamination, virgin sterile scalpel blades were used for each membrane filter. The eDNA was isolated using the Zymo Research (Irvine, CA, United States) Quick-DNA Fecal/Soil Microbe Miniprep Kit (Catalog number D6010). As a control, membranes were wetted with 100 μL of the kit’s elution buffer and processed like the other filters. Cell disruption and lysis were performed by placing membrane fragments into the kit’s lysis tubes. A Bead Bug Homogenizer (Benchmark Scientific, Sayreville, NJ, United States) shaken at 4,000 cycles per minute was used for 180 s. The manufacturer’s instructions were followed for the remaining DNA purification steps. To remove contaminating RNA, isolated DNA was treated with 1/10 volume of 10 mg/mL RNase A (37°C for 30 min). The DNA was purified and size selected (>500 pb) using 0.65X volume of Mag-Bind Total Pure NGS magnetic beads (Omega Bio-tek, Norcross, GA, United States) per the manufacturer’s instructions. DNA purity was assessed by measuring the 260 nm/280 nm optical density (OD) absorption ratio with a NanoDrop Lite Spectrophotometer (ThermoFisher, Waltham, MA, United States). All samples had an OD260/280 ratio of less than 1.9. DNA concentrations were measured with an Invitrogen Qubit fluorimeter (ThermoFisher; Double Stranded DNA Broad Range Assay Kit, Catalog number Q32853). The size of the RNase A treated eDNA was evaluated using a rapid gel electrophoresis system (1.2% DNA FlashGel, Lonza Group, Basel, Switzerland).

### 16S Amplicon Library Construction and Sequencing

Library preparations and sequencing were performed by a commercial service (Molecular Research Laboratory, Shallowater, TX, United States). The 16S rRNA gene variable region V4 (Gray et al., 1984) was amplified using Illumina (San Diego, CA, United States) barcoded oligonucleotides that contain the priming sequences 515F-GTGYCAGCMGCCGCGGTAA (Parada et al., 2016) and 806R-GGACTACNVGGGTWTCTAAT (Apprill et al., 2015). Polymerase chain reaction (PCR) was performed using the HotStarTaq Plus Master Mix Kit (Qiagen, Hilden, Germany). The thermocycling protocol was as follows: polymerase activation by heating at 94°C for 3 min; 28 cycles of melting at 94°C for 30 s; annealing at 53°C for 40 s; and primer extension at 72°C for 1 min. An additional elongation step of 72°C for 5 min was added to the last cycle. After 2% agarose gel electrophoresis, successfully produced amplicons were pooled in equal molar amounts and purified using Ampure XP beads (Beckman Coulter Life Sciences, Indianapolis, IN, United States). The library was sequenced with an Illumina MiSeq using the manufacturer’s protocol. After sequencing, barcodes were removed. Sequences shorter than 150 pb were purged, and chimeras were removed. Ten of ten samples were successfully sequenced from the winter samples while nine of ten samples were successfully sequenced from the summer samples.

### Analysis Pipeline

**FIGURE 2:**
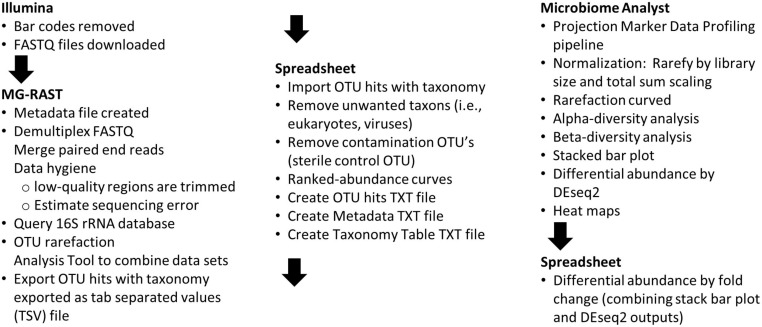
Flow-chart of data analysis steps. The order of activities and the software tools used to accomplish the corresponding tasks are described.

To facilitate data processing by undergraduates with no command-line computing experience, software pipelines with web-based graphical user interfaces (GUI) were used. A flow-chart of the data analysis steps used is shown in Figure 2 and a detailed description of how the students completed the steps is presented in Supplementary Table 1. Metagenomics Rapid Annotation using Subsystem Technology (MG-RAST; Meyer et al., 2008) version 4.0.3 (Argonne National Laboratory, 2017) web-based pipeline was used as a sequence data repository, to perform data quality control, and to query the 16S rRNA databases (Quast et al., 2012).

Students in the 2020 course performed data analysis by accessing the web-based MicrobiomeAnalyst pipeline (Dhariwal et al., 2017). After data uploading, the students used the Projection with Marker Data Profiling (PPD) pipeline. To deal with data paucity in low abundance taxa (Weiss et al., 2017), a filter was used to remove OTUs with fewer than four counts in 20% of the data cells. After filtering, 341 OTU’s were assessed. To deal with variability in library sizes, the data was rarefied without replacement to the minimum library size. The data was normalized by total sum scaling (Weiss et al., 2017). The pipeline was used to analyze the data with rarefaction curves, alpha-diversity tools, and beta-diversity tools. Additionally, differential abundance was evaluated by using built-in RNAseq tools [DEseq2 algorithm (Love et al., 2014)]. An MA-plot (Love et al., 2014) was created by using a spreadsheet to merge the log2-fold change data (M) calculated by DEseq2 with average OTU count data (A). The larger abundance average (summer versus winter) was used to plot the *A*-axis.

## Results

### Limnology

To evaluate the trophic status of the study site, nutrient concentrations of the water and benthic sediments were measured (Table 1). Notably high concentrations of orthophosphate were observed in the water. Additionally, high levels of phosphorous were detected in the sediments.

**TABLE 1:** Nutrient data.

Water chemistry (Winter 2019)						
Parameter	Sample size	Mean	Standard deviation	95% confidence interval		Units
Orthophosphate	9	31.2	11.0	39.6	22.8	mg/L
Hardness, Ca^2+^	9	98.0	4.6	101.5	94.5	mg/L
Ammonium	3	0.23	0.09	0.46	0.01	mg/L
Nitrate	9	3.3	2.2	5.0	1.5	mg/L
_P_H	9	6.70	0.27	6.90	6.49	

**Sediments (Summer 2018)**						

**Parameter**	**Sample** **size**	**Mean**	**Standard deviation**	**Units**		

Phosphorous	3	224	0	kg/ha		
Potassium	3	477	0	kg/ha		
Nitrogen	3	17	0	kg/ha		
pH	3	7	0			

### 16S Metagenomic Libraries

The students were successful in isolating high quality eDNA from bacteria sampled from the frozen cove. After RNaseA treatment, the DNA had a modal size of >4 kbp (Supplementary Figure 1A) and was successfully used to create libraries containing 16S rRNA encoding amplicons. After Illumina MiSeq sequencing, the SILVA 16S rRNA gene database was queried by the students. The hits observed from the libraries ranged from 14,995 hits to 178,120 hits, with the median being 62,317 hits.

**FIGURE 3:**
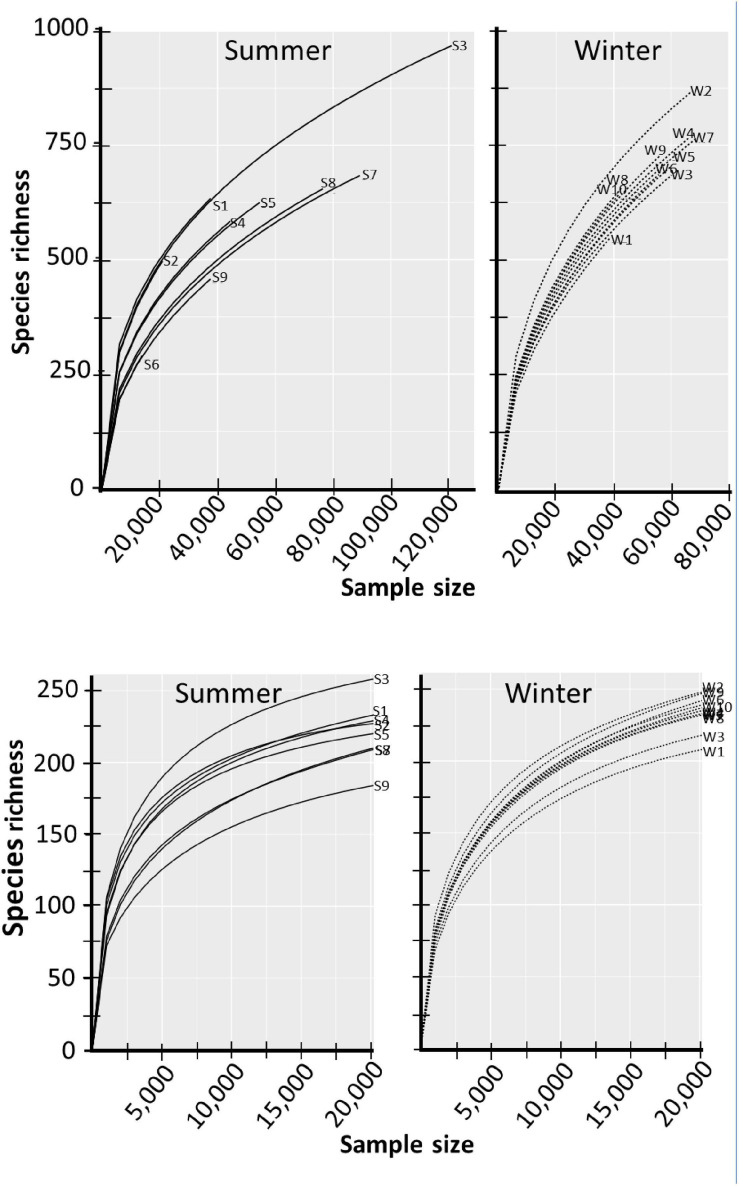
Species curves of unaltered and filtered-rarefied datasets. Raw OTU counts that represent species richness are presented in the top two panels. The filtered and rarefied datasets are presented in the bottom two panels. Libraries from summer-collected samples are labeled with S and winter-collected samples with W.

Species accumulation curves were used to determine if the sequenced libraries were representative of the species richness of the prokaryotic communities (Figure 3). In both the summer samples and the winter samples, the slopes of the curves of the low-count unfiltered datasets did not produce an asymptote, even library S3 that produced over 120,000 hits to the SILVA 16S rRNA database. The lack of an asymptote indicates that the libraries did not capture the complete species richness of the prokaryotic community. Additionally, the graphs show that the sequencing runs did not produce datasets of equal sampling efforts, especially the libraries made from the summer samples. As a result, the data sets were rarefied before subsequent analysis. The bottom panels of Figure 3 showed that the filtering and data rarefaction produced datasets representing equal sampling efforts, making the data amenable to statistical analysis.

### α-Diversity

**FIGURE 4:**
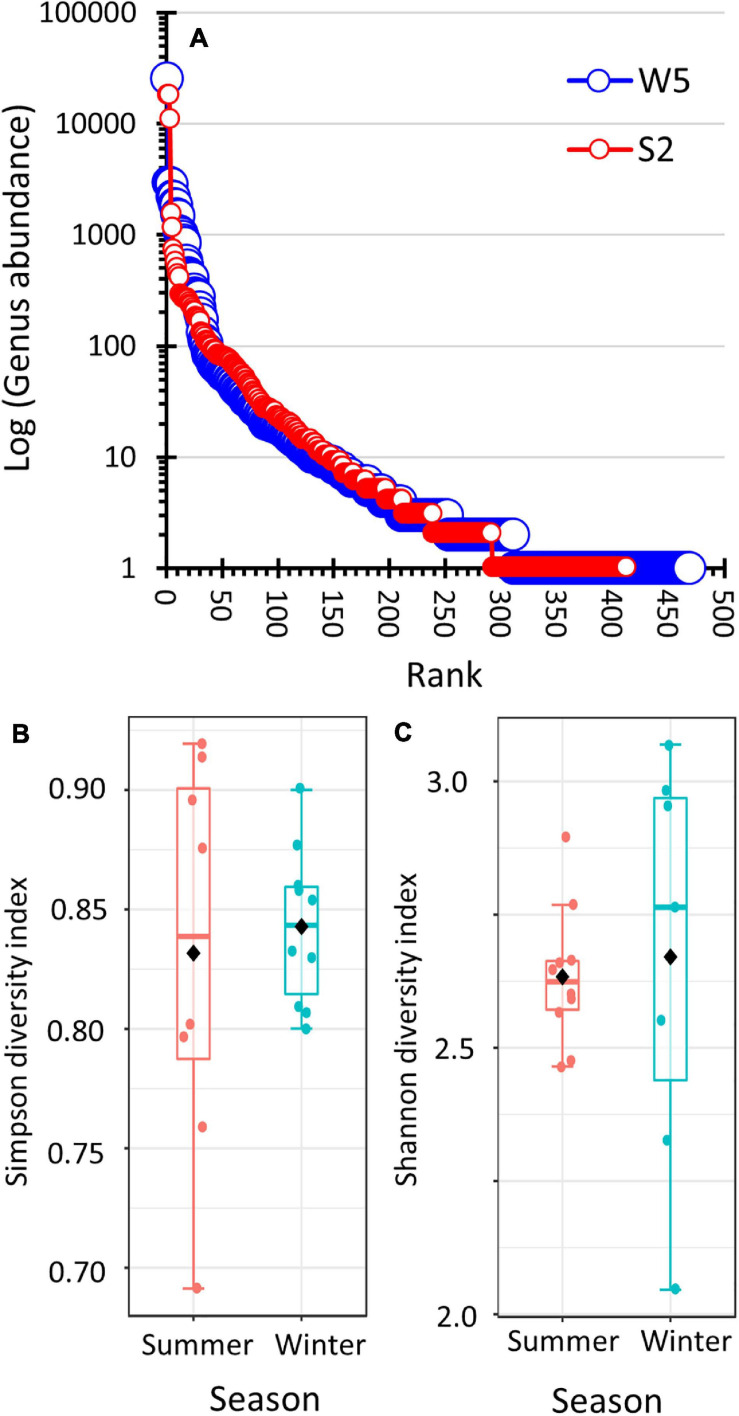
Comparison of the alpha-diversity of summer and winter prokaryote populations. **(A)** Ranked-abundance curve by genera of summer sample (S2) and winter sample (W5). Both libraries produced nearly identical sampling efforts (62,317 hits and 63,700 hits to the SILVA 16S rRNA database, respectively). The S2 data was normalized to 63,700 hits by multiplying the hit count for each genus by 1.022. **(B,C)** Box-and-whisker plots comparing Simpson diversity and Shannon diversity, respectively, of the summer versus winter prokaryote populations. The data set was filtered and rarefied.

The students used three methods to compare the α-diversity (i.e., taxonomic diversity within a habitat) of the winter and summer prokaryote communities. Unfiltered data was used to produce ranked abundance curves. Summer versus winter data sets of nearly equal sizes were compared (Figure 4A and Supplementary Figures 1B,C). The analysis showed that both the summer and winter bacteria populations produced nearly identical genera abundance structure. Even on a log-scale, the distribution produced a steep negative-sloping curve. Analysis using Simpson’s diversity index indicated that both communities showed similar genera diversity (Figure 4B). The difference in the diversity indices was not statistically significant (two-sample *t*-test assuming unequal variance: winter *x̄* = 0.8349, *s* = 0.0367; *x̄* = 0.7941 and *s* = 0.1135; pooled degrees of freedom = 8, *t* = 0.9754, *p* = 0.3579; and Shapiro Wilk test of normality: winter *p* = 0.9712, summer *p* = 0.2081). Similar results were obtained when using Shannon’s diversity index (Figure 4C, two-sample *t*-test assuming unequal variance: winter *x̄* = 2.633, *s* = 0.1292; summer *x̄* = 2.650, *s* = 0.3803; pooled degrees of freedom = 7, *t* = −0.2495, *p* = 0.8101; and Shapiro Wilk test of normality: winter *p* = 0.5777, summer *p* = 0.5215).

### β-Diversity

**FIGURE 5:**
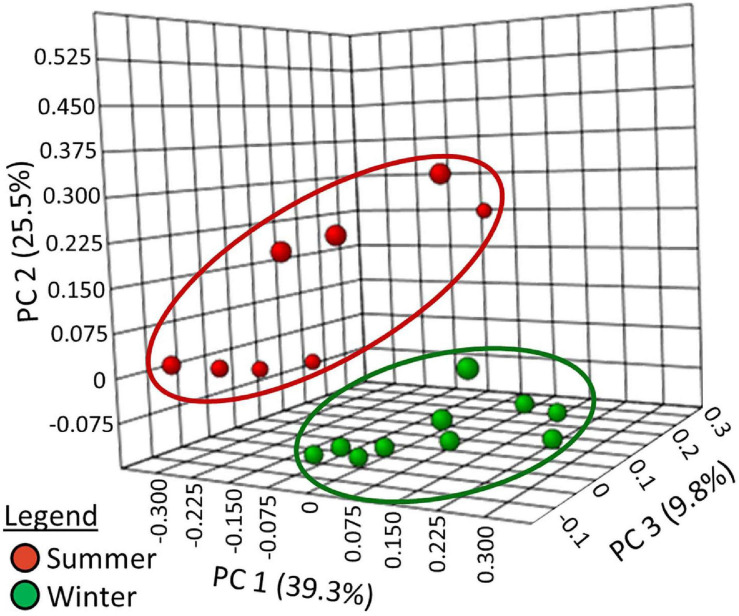
Principal coordinates analysis of Bray–Curtis Index distance measurements at the taxonomic level of the genera.

To evaluate β-diversity (i.e., comparison of taxonomic diversity between habitats), the students used principal coordinate analysis of Bray–Curtis dissimilarity indexes (Figure 5). The summer samples and winter samples produced two distinct clusters. ANOSIM showed the clustering to be statistically significant (*r* = 0.59829, *p* < 0.001). To determine which phyla were responsible for the observed differences in β-diversity, changes in abundance were analyzed.

**FIGURE 6:**
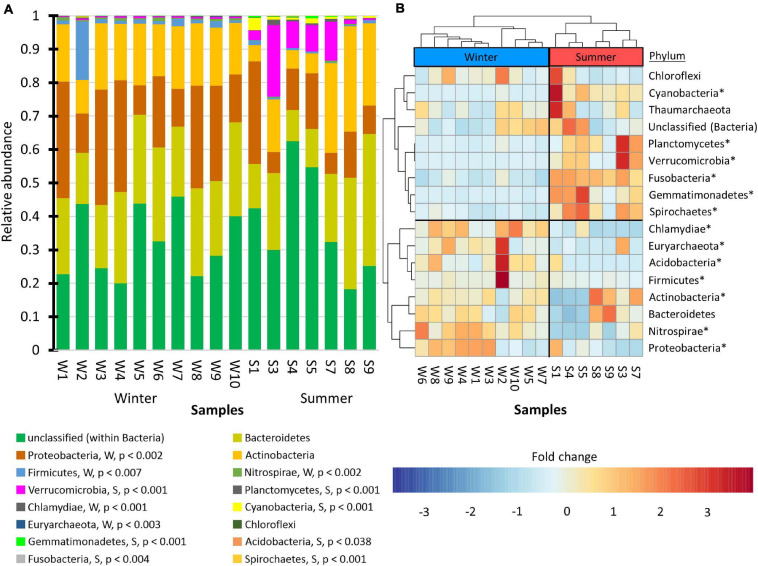
Changes in prokaryote abundance by phyla. **(A)** Stacked bar chart of relative abundance. The phyla were arranged from greatest mean winter abundance to lowest mean winter abundance. DEseq2, with a false discovery rate set at 0.05, was used to assess the statistical significance of the fold changes. Phyla that showed greater winter abundance are indicated with a “W” while those with greater summer abundance are indicated with “S.” The adjusted *p*-values are shown. **(B)** Dendrogram with heat-maps that were hierarchically clustered by average Pearson correlation coefficient. Phyla that were statistically significant in **(A)** are marked with asterisks in **(B)**.

To visualize which phyla were associated with winter versus summer communities, the students created stacked bar charts (Figure 6A). The graph shows that the majority of the observed phyla were present in low abundance. Only one phylum, Proteobacteria, was highly prevalent and showed increased abundance in the winter. Additionally, only one phylum, Verrucomicrobia, was highly prevalent and showed increased abundance in the summer. Dendrograms with differential abundance heat-maps (Figure 6B) produced a distinct summer clade and a winter clade. Similar results were also produced when taxonomic orders were analyzed (Supplementary Figure 1D).

**FIGURE 7:**
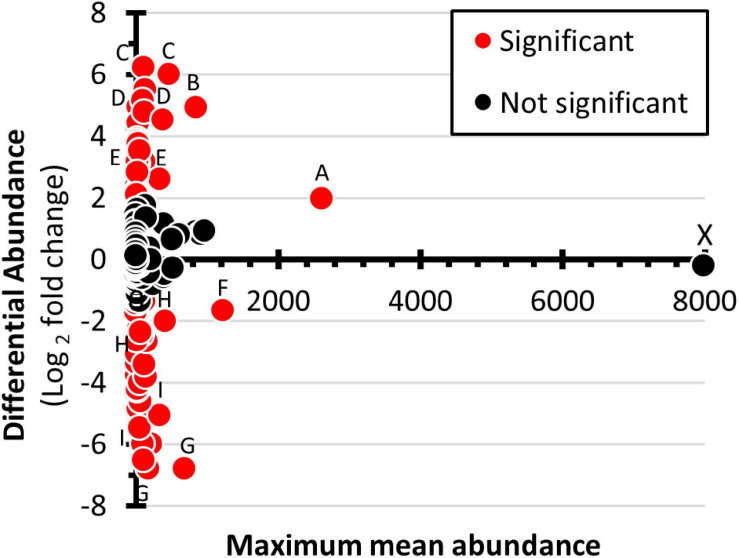
Differential abundance of prokaryote genera. Maximum mean abundance (summer versus winter) is presented on the *X*-axis. Differential abundance expressed as log_2_ [(mean winter abundance) – (mean summer abundance)] is presented on the *Y*-axis. Genera with greater winter relative abundance are given positive values and those with greater summer abundance are given negative values. Red data points represent genera that have statistically significant change in abundance, as determined by DEseq2, with a false discovery rate set at 0.05. The labeled data points correspond to the following genera: A, unclassified within Betaproteobacteria; B, *Prolixibacter*; C, unclassified within the Sphingobacteriaceae; D, *Delftia*; E, *Clostridium*; F, *Cryobacterium*; G, *Rubritalea*; H, *Terrimonas*; I, *Chthoniobacter*; and X, unidentified phylum in Bacteria domain.

An MA-plot (Figure 7) was used to show the differential abundance of genera. Of the 230 genera in the analysis, 80 had greater abundance in the winter samples and 59 were more abundant in the summer samples (Supplementary Table 2). Six genera showed substantially increased winter abundance: unclassified within Betaproteobacteria, *Prolixibacter*, unclassified within the *Sphingobacteriaceae*, *Delftia*, and *Pedobacter* (descending order). Five genera showed substantially increased summer abundance: *Clostridium*, *Cryobacterium*, *Rubritalea*, unclassified within the Gamaproteobacteria, *Terrimonas*, and *Chthoniobacter*.

### Course Assessment

**FIGURE 8:**
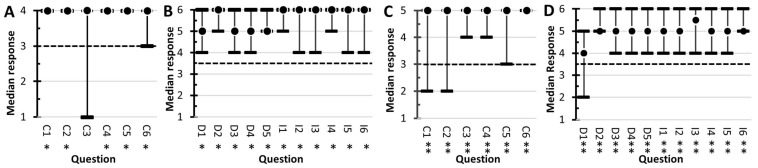
Student responses to the Laboratory Course Assessment Survey. Results from the 2019 survey are shown in **(A,B)**. The 2020 results are in **(C,D)**. The dot represents the students’ median response. The range bars are the range of responses. The dotted line corresponds to the null hypothesis used in the sign-tests. All responses statistically significant at α = 0.05 are marked with single asterisks (^∗^) and significance at α = 0.01 are marked with a double asterisk (^∗∗^). The questions for the Collaboration section **(A,C)** started with, In this course, I was encouraged to, and ended with, (C1) discuss elements of my investigation with classmates or instructors, (C2) reflect on what I was learning, (C3) contribute my ideas and suggestions during class discussions, (C4) help other students collect or analyze data, (C5) provide constructive criticism to classmates and challenge each other’s interpretations, and (C6) share the problems I encountered during my investigation and seek input on how to address them. The answer options for the 2019 survey **(A)** were never (1), one or two times (2), monthly (3), and weekly (4). In the 2020 survey **(C)**, the options were never (1), one or two times (2), monthly (3), every other week (5), and weekly (6). The Discovery and Relevance questions **(B,D)** started with, In this course, I was expected to, and ended with (D1) generate novel results that are unknown to the instructor and that could be of interest to the broader scientific community or others outside the class, (D2) conduct an investigation to find something previously unknown to myself, other students, and the instructor, (D3) formulate my own research question or hypothesis to guide an investigation, (D4) develop new arguments based on data, and (D5) explain how my work has resulted in new scientific knowledge. The Iteration section questions started with, In this course, I had time to, and ended with, (I1) revise or repeat work to account for errors or fix problems, (I2) change the methods of the investigation if it was not unfolding as predicted, (I3) share and compare data with other students, (I4) collect and analyze additional data to address new questions or further test hypotheses that arose during the investigation, (I5) revise or repeat analyses based on feedback, and (I6) revise drafts of papers or presentations about my investigation based on feedback. The answer options were, strongly disagree (1), disagree (2), somewhat disagree (3), somewhat agree (4), agree (5), and strongly agree (6).

Students’ perceived experiences in conducting authentic research were assessed using the LCAS (Figure 8). The Collaboration component of the LCAS assesses the frequency that collaborative activities occurred during the course. Two-tail sign-tests were used to assess the null hypothesis that Collaborative activities occurred monthly. In 2019 (Figure 8A), responses to questions C1, C2, C4, C5, and C6 were statistically significant (*p* < 0.05). The results indicated that collaborative activities were perceived to occur more frequently than monthly, with the median response corresponding to weekly. The null hypothesis was accepted for C3 (6 positives, 1 negative, and *p* = 0.1250). In 2020 (Figure 8C), responses to all the Collaboration questions were statistically significant (*p* < 0.01). The results indicated that the students’ perceived collaborative activities more frequently than monthly, with the median value being weekly.

The Discovery section of the LCAS (Figures 8B,D) assesses students’ perceptions of their experiments contributing to new scientific knowledge. The Iteration section assesses student perceptions of the frequency that procedures were duplicated and the frequency that experiments were repeated to resolve problems with their data. Both sections used a six-point Likert scale. The students’ responses were evaluated with two-tail sign-tests, using the null hypothesis median = 3.5. For the 2019 class, all the Discovery questions and Iteration questions produced statistically significant responses (*p* < 0.05). For the 2020 class, all the Discovery questions and Iteration questions produced statically significant responses at *p* < 0.001. The results indicated that the students perceived that they participated in iteration-processes associated with the scientific method and their research activities were scientifically relevant.

**FIGURE 9:**
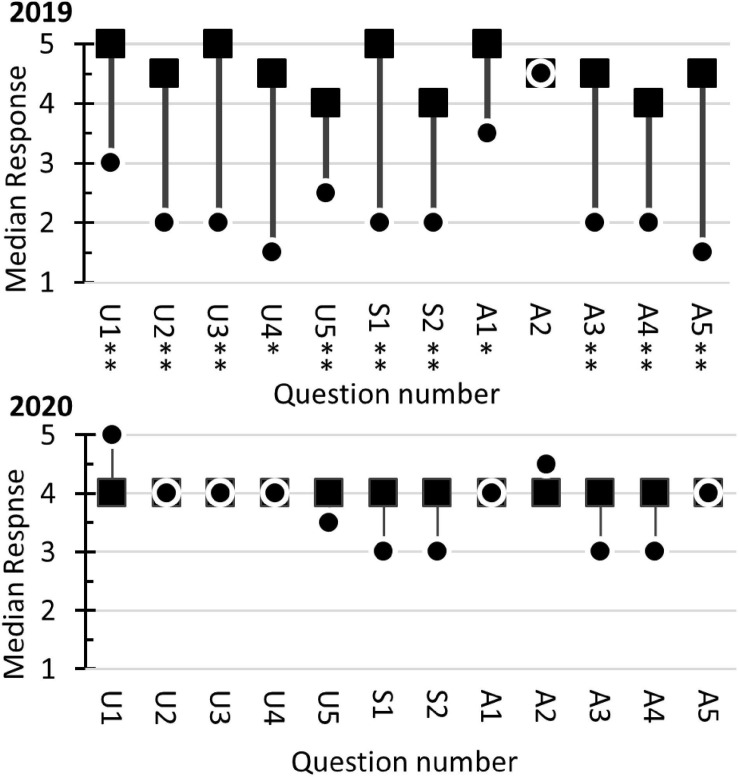
Student responses to the GCAT-SEEK opinion survey in 2019 and 2020. The survey used a Likert scale response system (1 “not at all” to 5 “a great deal”). The dots (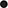) represent the students’ median response in the pre-course survey. The squares (■) indicate the students’ median response in the post-course survey. A two-tailed Mann–Whitney *U*-test for two independent samples was used to assess the null hypothesis Median_*Pre–course*_ = Median_*Post–course*_. Statistically significant differences at α = 0.05 and α = 0.01 are marked with single and double asterisks, respectively. The questions related to student perception of their Understanding (U) started with the phrase, Presently, I understand, and ended with U1, the genetic mechanisms that underlie evolution (mutation, selection, migration, drift, and etcetera.); U2, the relationship between basic units of molecular structure and their function; U3, how bioinformatics can be used to understand the flow, exchange, and storage of information from genome to phenotype; U4, how the genome confers metabolic capabilities to an organism; and U5, how genomic analysis can elucidate larger scale interactions within organisms, between organisms, and/or between organisms and ecosystems. The Skills (S) questions assess students’ perception of their abilities and started with the phrase, Presently, I can, and ended with S1, identify patterns in bioinformatics data; S2, recognize a sound argument based on the appropriate use of bioinformatics evidence. The Attitudes (A) questions started with the phrase, Presently, I am, and ended with A1, enthusiastic about next-generation sequencing; A2, interested, if the opportunity is available, in taking further courses/performing more research in this topic area; A3, confident that I understand next-generation sequencing technologies; A4, confident that I can analyze next-generation sequencing data; and A5, confident that I can incorporate next-generation sequencing technologies into my research.

In addition to the LCAS, a survey created by GCAT-SEEK was used to evaluate the students’ attitudes and perceptions related to next-generation sequencing. The results from the 2019 course (Figure 9) indicated that the students felt their understanding of genetics, biochemistry, and bioinformatics increased after completing the course. Analyses of the Understanding questions with Mann–Whitney *U*-tests detected statically significant (*p* < 0.05) increases of median response scores for all questions. Additionally, the two questions related to students’ bioinformatics skills showed statistically significant increases. The students also showed a statistically significant increase in their “enthusiasm” regarding next-generation sequencing (question A1). They also indicated increased confidence (questions A3 to A5) in their ability to use next-generation sequencing in future research. There was no change in students’ interest in taking additional courses (question A2), possibly because their initial interest was already high (median = 4.5 on a 5-point scale). Students answering the questionnaire in 2020 reported high scores in all categories of the questionnaire. As a result, no statistically significant changes were observed in the pre-course/post-course median responses. Comparisons of pre-course questionnaire responses from 2019 to 2020 showed the 2020 students had statistically greater median scores (*p* < 0.05) for questions U1 to U4, S1, S2, and A5. The results indicated that the 2020 students felt they had a greater understanding of the concepts and better analytical skills at the beginning of the course than their 2019 counterparts.

For qualitative assessment, an anonymous end-of-course student questionnaire was given to the students. Their verbatim responses are presented in Supplementary Table 3. In 2019 and 2020, students’ responses to the question, “What aspects of the course did you like?” were longer than their responses to “What changes can be made to improve the course.” Some noteworthy comments made in 2019 related to the students’ positive attitudes toward field collections during the winter. Because the course switched to an online format due to the COVID-19 epidemic in 2020, those students did not have the opportunity to participate in field collection. A theme observed in both the 2019 and 2020 surveys was student comments on the hands-on nature of their experience, collaborations with their peers, repeating procedures that did not work in the first attempt and performing experiments where the answers were not already known. Some students noted that having cycles of draft and revision of their laboratory reports was beneficial to their learning.

## Discussion

### Students Conducted Authentic Research

Undergraduates enrolled in the CURE course Applied Metagenomics were successful in conducting an authentic scientific investigation. The students’ chemical analysis of the water samples (Table 1) showed high orthophosphate concentrations. As a result, the cove-water can be classified as eutrophic (Carlson, 1977). The presence of dense mats of three duckweed species (Baker, 2018) also indicates that the water is eutrophic (Landesman et al., 2011). The likely source of nutrients is the Clinton River Bypass (Figure 1), a waterway high in nutrient and sediment pollution (Healy et al., 2008). Sediments from the bypass can be observed entering the cove (personal observation). Analysis of the benthic sediments collected from the cove showed high nutrient levels, including phosphorous (Table 1).

The students used some standard computational approaches(Gotelli and Chao, 2013) to evaluate community diversity. Species accumulation curves (Figure 3) indicated that the 16S rRNA sequence data sets did not sample all the species present in the summer and winter samples. The largest sequencing library (S3) detected 1,032 OTUs (Supplementary Table 2). Because detected species richness is a function of sampling effort (Gotelli and Chao, 2013) and the libraries had over a 10-fold difference in sequencing depth (Weiss et al., 2017), the students conducted most of the subsequent data analysis with rarefied datasets.

Multiple approaches were used to evaluate α-diversity by the students. One approach was to use spreadsheets to create ranked abundance curves (Smith and Smith, 2015) using data from libraries of equal sequencing depth (Figure 4A and Supplementary Figures 1B,C). Since 16S rRNA barcoding cannot reliably classify bacteria to the species level (Lebonah et al., 2014), the ranked abundance curves were created at the genera level as defined by the SILVA 16S rRNA databases (Quast et al., 2012). The graphs had backwards-J shapes indicating the communities were comprized of one to three highly abundant genera. The winter and summer lines on the graphs overlapped, which indicated that the amount of prokaryote diversity in the winter samples was the same as in the summer samples. This conclusion was supported by calculating diversity indices. Simpson’s and Shannon’s diversity indices measure diversity by considering the number of taxa and the evenness of distribution of the taxa (Smith and Smith, 2015; Kim et al., 2017). The box-and-whisker plots of both diversity indices overlapped, thus showing no difference in α-diversity between summer and winter samples.

The students used the Bray–Curtis dissimilarity index to evaluate β-diversity. This index was chosen because it is the complement of the Sørensen similarity index, a community comparison index presented in many undergraduate ecology textbooks (Smith and Smith, 2015). Principle coordinate analysis (Figure 5) showed that the winter and summer community compositions were distinct. Dendrograms with heat-maps were used to display the differential abundance of phyla (Figure 6) and orders (Supplementary Figure 1D). The data clearly showed that the taxonomic composition of the winter prokaryotic community was different than that of the summer community.

The community compositions of the cove (Figure 6) contained the same phyla identified as ubiquitous freshwater bacteria by Zwart et al. (2002) and Newton et al. (2011). They are Proteobacteria, Actinobacteria, Bacteroidetes, Cyanobacteria, Verrucomicrobia. As a result, the students concluded that the composition of the prokaryote community in the cove was typical of freshwater ecosystems.

In contrast, the students concluded that the composition of the frozen cove community was unlike communities in frozen tundra lakes described by Vigneron et al. (2019). When frozen, the Methanogens, Planctomycetes, Chloroflexi, and Deltaproteobacteria became abundant in tundra lakes. In contrast, no Methanogens or Deltaproteobacteria in any of the lake samples were observed by the students (Figure 6). The undergraduates did observe Chloroflexi and Planctomycetes, but they were more abundant in the summer samples. In the summer, Actinobacteria and Betaproteobacteria were the predominant phyla in the tundra lake. In contrast, in the Lake Saint Clair samples, Betaproteobacteria were not predominant, and Actinobacteria were more abundant in the winter samples. These results indicated that the community composition of the eutrophic temperate water was distinctly different than the community composition observed in a tundra lake.

The most abundant phylum detected by the students in all water samples was classified as unidentified (Figure 6). This phylum contained a single OTU (Supplementary Table 2). Thus, this organism likely has not been described by science. OTU2675 represented 31% of the counts in the dataset. Although it is the most prevalent bacterium in the community, it likely does not grow on tryptic soy agar or minimal media. Over 30 bacteria strains have been isolated as pure cultures by students taking an ecology laboratory course. 16S rRNA barcodes of these isolates did not correspond to OTU2675 (personal observation). The probability of obtaining this result due solely to chance is 1.46 × 10^–5^. This result showed the students that culture-based methods can miss environmentally prominent organisms, thus illustrating one of the strengths of using metagenomics to study microbial ecology.

Because of their great metabolic diversity, it is difficult todetermine the ecological role of prokaryotes by just evaluating higher-level taxa. Thus, differential abundance level was analyzed at the level of genera. With 224 genera in the data set (Supplementary Table 2), stacked bar charts and hierarchically arranged heat-maps were inadequate methods of presenting the data. To solve the problem, the students used spreadsheet software to create an MA plot to analyze differential abundance at the level of the genera (Figure 7). Four genera stood out as having increased abundance in the winter samples; unclassified within Betaproteobacteria; *Prolixibacter*; unclassified within the Sphingobacteriaceae; *Delftia*; and *Clostridium*. Four genera showed prominently increased abundance in the summer samples; *Cryobacterium*, *Rubritalea*, *Terrimonas*, and *Chthoniobacter*. The most abundant genera corresponded to the unidentified OTU2675 bacteria. Its relative abundance was nearly identical in winter and summer samples. Based on its position on the *A*-axis, this bacterium was the dominant prokaryote in the cove. One possible line of future student investigation is to determine the prevalence of the species in other locations within Lake Saint Clair and other waterways of the Great Lakes Region.

Analyzing the natural history of prominent genera may provide insights into the ecology of the frozen lake and become a basis for students to develop testable hypotheses. For example, datapoint-A (Figure 7) corresponds to an unclassified genus within Betaproteobacteria. Betaproteobacteria are often numerically dominant in lake epilimnia, have rapid growth rates, are major components in microbial grazing food chains, and prefer nutrient-rich environments (Newton et al., 2011). Thus, organism-A may have increased its relative winter abundance due to the exploitation of winter-abundant resources. Another example is the genera *Prolixibacter* (datapoint-B). Members of this taxon are non-cellulosic fermenting facultative anaerobes that have been isolated from marine sediments (Holmes et al., 2007) and cold (5°C) peat bogs (Schmidt et al., 2015). Often, biological oxygen demands cause hypoxia in ice-covered lakes (Ellis and Stefan, 1989). Thus, the increased prevalence of *Prolixibacter* may be due to its being adapted to cold low oxygen environments. To test this hypothesis, dissolved oxygen measurements can be conducted of water samples collected from under the ice.

Nutrient availability may be a factor causing an increased abundance of some genera. For example, *Delftia* abundance increased 32-fold in the winter samples. The two corresponding OTUs had high homology to *D*. *acidovorans* and *D*. *tsuruhatensis*. The type specimens for these species were isolated from high nutrient environments (Han et al., 2005; Yilmaz and Icgen, 2014). Another genus, *Clostridium*, had a 6-fold greater prevalence in the winter samples. Members of this genera have been isolated from activated sludge (Gumaelius et al., 2001). Many strains are aerobic denitrifiers. The presence of *Clostridium* suggests an active role in nutrient turnover.

The pattern observed in one of the differentially abundant genera ispuzzling. *Cryobacterium*, represented by a single OTU, showed a 2.8-fold increased abundance in the summer samples (Figure 7, datapoint F). The *Cryobacterium* OTU had high homology to *C. psychrophilum* and was the 2nd most abundant OTU in the summer dataset (Supplementary Table 2). The type specimen of *C. psychrophilum* was isolated from samples in Iceland. It grew best in cool water (9 to 12°C) and stopped growing when the temperature reached 18°C (Suzuki et al., 1997). When the water samples were collected in the summer, the surface temperature was 23°C. Thus, the increased prevalence of the *C. psychrophilum*-like bacteria in the summer sample is unexplained and warrants further investigation.

### Student Data Analysis Workflow

One of the goals in the development of the Applied Metagenomics CURE course was to overcome computing-barriers in processing metagenomics data. The data presented in this manuscript show that undergraduates without knowledge of computer coding or command-line computing can complete a metabarcoding investigation. However, the students did find some of the computing tasks difficult to accomplish. The nature of the difficulties and strategies used to overcome the bottlenecks are presented in Supplementary Table 4.

The approach of using MG-RAST in combination with MicrobiomeAnalyst can be used to analyze shotgun metagenomic sequence data as well since both portals support this type of data. Additionally, undergraduates can use other pipelines to analyze metabarcoding data sets. Recently, CyVerse has beta-released the Purple Line of its DNA Subway (CyVerse, 2019), a GUI-based version of the QIIME 2 pipeline (Bolyen et al., 2019). As a result, students can use more than one approach to process 16S rRNA metabarcoding data.

### Limitations When Using CUREs

Though CUREs can contribute to scientific knowledge, there are inherent limitations on the nature of the investigations that can be conducted. For example, undergraduate students do not have access to the array of resources often available in research laboratories. In this course, the students wanted to obtain water samples that were as representative of the prokaryotic community as possible. However, they did not have access to a mobile field laboratory to perform immediate microbial isolation. Though the collection vessels were filled to the lip, closed with an air-tight cap, and kept on wet-ice for less than 2 h, some organisms, such as obligate anaerobes, may have been lost. Many other metagenomic investigations of environmental water samples have stored samples on wet-ice before microbe isolation (Yannarell et al., 2003; Shade et al., 2007; Van Rossum et al., 2015; Uyaguari-Diaz et al., 2016; Linz et al., 2017; Karayanni et al., 2019; Mohiuddin et al., 2019). Thus, the collection procedure that were used by the students is within the norms of basic research.

Another limitation to CURE studies is the timeframe of the investigation. Ideally, a longitudinal study like this one would be conducted over consecutive seasons and multiple years. However, the CURE course only lasted one semester (15 weeks). The students were able to compare different seasons because they were able to utilize a data set created 3 years earlier. Though the primary conclusions are valid (i.e., the microbial community from the ice cover lake samples were as diverse as the open water summer samples, and the compositions of the two communities were strikingly different), the students could not determine the variability of the community structure from one year to the next. Finally, budget constraints limit the number of samples analyzed. For this course, the maximum number of samples, including controls, that could be used in the experimental design was limited to 12 sequencing runs.

### Course Assessment

The course was assessed to determine if the goals of a typical CURE were accomplished. The LCAS (Corwin et al., 2015) is designed to measure three attributes of CUREs. Students were asked six questions regarding their perceptions of collaborative activity frequency. The results in Figure 8 showed that the students felt that they discussed with their peers or the instructor elements of their investigations, reflected on their learning, contributed to discussions, collaborated on data analysis, and collaborated on resolving problems on a weekly basis.

Five questions on the LCAS evaluated the students’ perceptions of their research as they relate to scientific discovery and scientific relevance. All questions produced statistically significant responses (*p* < 0.05 in 2019; *p* < 0.01 in 2020) to the null hypothesis of neutral attitude (i.e., Median = 3.5). The lowest median response observed was to question D1, generating novel results unknown to the instructor or scientific community. The lead author (SSB) was surprised by this result because the discovery-nature of the course was explicitly conveyed to the students. In contrast, questions addressing students’ perception of their investigating something previously unknown (D2), formulating a hypothesis (D3), developing an argument based on evidence (D4), and creating new scientific knowledge (D5), the median responses were “highly agree” or “agree.” To resolve the dichotomy in the students’ attitudes, open-response questions will need to be added to future surveys. In total, the students’ responses to this section of the LCAS indicate that the students felt their research contributed to scientific knowledge and was scientifically relevant.

Six LCAS questions evaluated the iteration processes used in scientific investigations. All questions produced statistically significant responses (*p* < 0.05 in 2019; *p* < 0.01 in 2020) with the median responses corresponding to “agree” or to “strongly agree.” These results indicate that the students felt they repeated work to fix problems with their results, changed methods in response to unanticipated results, compared their results to the results of their peers, collected additional data to help revise hypotheses, responded to feedback from others, and revised their written work.

The GCAT-SEEK opinion questionnaire was used to assess students’ attitudes to next-generation sequencing technologies (Figure 9). In 2019, students reported an increased understanding of the genetic mechanisms related to evolution, the relationships of molecular structure and functions, genome information flow, and how genomes control metabolism (*p* < 0.05). The same students felt their skills in using bioinformatics to identify patterns and making arguments increased after completing the course. The students also indicated a more positive attitude toward research involving next-generation sequencing. The median “enthusiasm” (A1) response increased from 3.5 (a neutral value) to 5 (highly agree). They also reported increased confidence in understanding (A3) and using (A4 and A5) next-generation sequencing data. The students indicated they had a “high” (median = 4.5) interest in performing more research with next-generation sequencing at the start of the course and maintained this interest after the course (*p* = 0.610).

A different response pattern was observed in the 2020 data. The students indicated they had a strong understanding of core concepts (U1 to U5) in the pre-survey, with a median value corresponding to “agree” or “strongly agree.” They maintained this opinion after they completed the course [*p* = 0.056 (due to a small increase in the post-survey) to 0.608]. The same patterns were observed with the skills questions (*p* = 0.082 to 0.110) and attitude questions (*p* = 0.154 to 0.984). The results indicate that the students maintained their positive attitudes regarding next-generation sequencing technologies after completing the course. Comparison of the pre-course responses of the 2019 and 2020 classes showed the 2020 class reported higher median scores for the understanding questions and skill questions. The differences were statistically significant for questions A1 to A4 and S1 to S2. These results suggest that the students taking the course in 2020 felt more intellectually prepared for the coursework than did the students in 2019.

Qualitative assessment involved the instructor giving the students anonymous open-ended survey questions (Supplementary Table 3). Major themes observed in the student comments indicated that the course contained some of the major elements of CUREs (i.e., Scientific Process, Discovery, Collaboration, and Iteration). Their comments aligned well with the response observed in the LCAS survey (Figure 9). In terms of areas for improvement, some students felt that the open-ended nature of the laboratory was “disorganized,” and the procedures were too time intensive. In total, the responses in the open-ended survey indicated that the students found the CURE elements (Auchincloss et al., 2014) of the course helpful to their learning.

### Supporting the Goals of *Vision and Change*

*Vision and Change* is a joint policy statement of the American Association for the Advancement of Science, the National Academy of Sciences, and other organizations on how undergraduate biology curricula should be reformed during the 21st century (Bauerle et al., 2009). Because of the ever-expanding nature of science, *Vision and Change* calls for biology education to focus on a few key concepts, develop student investigative competencies and enhance student engagement in the scientific process (Woodin et al., 2010). The Applied Metagenomics course incorporates many of the *Vision and Change* recommendations. For example, students used the concept of evolution and biological information flow to analyze the results of their experiment. Additionally, the students developed competencies in using large data sets and computational analysis. Moreover, mathematical and communication skills were developed by having students write formal laboratory reports where they had to interpret their numeric data and clearly present their results with graphs. Finally, the students were fully engaged in the scientific process, because the research they performed was authentic and contributed to the knowledgebase of society.

The development of educational strategies that help retain undergraduate underrepresented minority students is identified as one of the “pressing needs” in *Vision and Change*. Large-scale studies of 6-year graduation rates showed that CUREs increase retention of underrepresented minority students (Jones et al., 2010; Schultz et al., 2011). CUREs may increase retention because the self-efficacy of underrepresented minorities increases when they participate in research (Hurtado et al., 2009). The results of the course assessment (Figures 8, 9) indicate that this metagenomic CURE course had a positive impact on the students’ attitudes toward research and thus has the potential of improving retention of underrepresented minority students.

## Conclusion

The instructional approach utilized in the Applied Metagenomics course can be used as a template to foster the development of additional CURE courses. The course was designed to overcome potential computational barriers (Maloney et al., 2010) by using publicly available web-based resources. Additionally, the data-analysis workflow used did not require students to learn command-line computing or programming. The students’ research was relevant because the sequence data was posted in a data repository and their research findings are published here. Additionally, the students’ data (i.e., posted sequence data and the OTU count data) can be used to develop additional *in-silico* activities for undergraduate instruction.

## Data Availability Statement

The datasets presented in this study can be found in online repositories. The name of the repository and accession numbers can be found in Supplementary Table 2.

## Ethics Statement

The studies involving human participants were reviewed and approved by University of Detroit Mercy’s Institutional Review Board (Protocol Number 1718-53). The patients/participants provided their written informed consent to participate in this study.

## Author Contributions

SB conceived of the project, created all instructional materials, confirmed all student calculations, created all figures, and wrote the manuscript. MA, KA, JC, GC, ZD, SK, ES, and SS collected environmental samples, isolated DNA, and did the primary data analysis with MG-RAST. ES, GC, ZD, NA, ZA-H, VC, DC, MH, MJ, MLJ, ZK, EK, RK, SK, AM, PP, RR, and ST performed additional data analysis with MicrobiomeAnalyst. All authors contributed to the article and approved the submitted version.

## Conflict of Interest

The authors declare that the research was conducted in the absence of any commercial or financial relationships that could be construed as a potential conflict of interest.

## Supplementary Material

Supplementary Figure 1 Supplemental student data.

Supplementary Table 1 Detailed description of the students’ data processing.

Supplementary Table 2 Prokaryotic count data and abundance analysis.

Supplementary Table 3 Results of anonymous end-of-course student questionnaire.

Supplementary Table 4 Overcoming bottlenecks in student workflow.
