# Supplementary material for: Students in a Course-Based Undergraduate Research Experience Course Discovered Dramatic Changes in the Bacterial Community Composition Between Summer and Winter Lake Samples
Source: Front Microbiol. 2021 Feb 18;12:579325. doi: 10.3389/fmicb.2021.579325 (PMC7929996; doi:10.3389/fmicb.2021.579325)
Supplement: Supplementary Figure 1 — Supplemental student data. [file Data_Sheet_1.pdf]

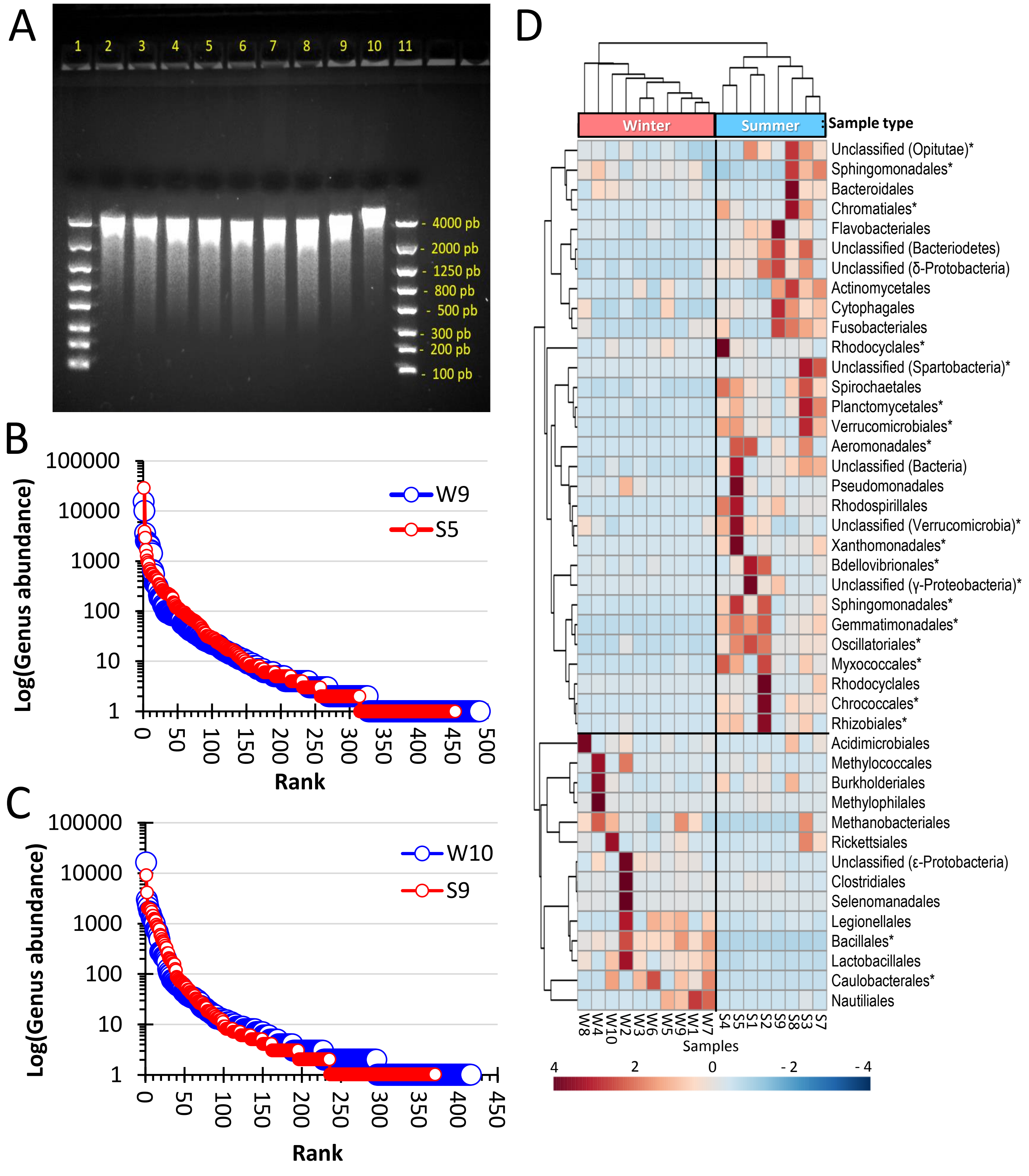

Figure S1: Supplemental student data. Panel A: Electrophoretic separation of eDNA. 100 ng of RNaseA treated DNA from winter water samples were loaded into lanes 2 though 10. Lanes 1 and 11 are DNA standards, whose size is shown on the right. Panels B and C: Ranked-abundance curves by genera of summer (S) sampled and winter (W) samples. Panel B, S5 had 59934 hits and W9 had 59977 hits. The S5 data was normalized to 59977 hits by multiplying the hit count for each genus by 1.0007. Panel C, S9 had 43402 hits and W10 had 44313 hits. The S9 data was normalized to 44313 hits by multiplying by 1.02099. Panel D: Heat-map of abundance by prokaryote orders hierarchically clustered by average Pearson correlation coefficient. 25% of OTU's with the smallest interquartile ranges were removed before clustering. Taxa marked with an asterisk (*) indicate the difference between the summer and winter relative abundance was statistically significant, as determined by DEseq2 with a false discovery rate set at 0.05.
