# Supplementary material for: Students in a Course-Based Undergraduate Research Experience Course Discovered Dramatic Changes in the Bacterial Community Composition Between Summer and Winter Lake Samples
Source: Front Microbiol. 2021 Feb 18;12:579325. doi: 10.3389/fmicb.2021.579325 (PMC7929996; doi:10.3389/fmicb.2021.579325)
Supplement: Supplementary Table 1 — Detailed description of the students’ data processing. [file Table_1.pdf]

**Table S1: Detailed description of the students' data-processing and bottlenecks.**

1) The students were asked to download the metadata Excel template from the MG-RAST upload page and view an online video tutorial (Argonne National Laboratory, 2012)

2) The two compressed (.qz) files containing the multiplexed read files (*.fastq), the multiplexing index file and the metadata file were uploaded to MG-RASTS as described in the video tutorial (Argonne National Laboratory, 2017).

3) To join pair-end reads the forward (R1) and reverse (R2) file icons were dragged into the "Drag file(s) here" box.

4) Sequences were demultiplexed by clicking the demultiplex icon and dragging the index file and the merged FASTQ sequence file into the "Drag file(s) here" box.

5) To submit the sequences for quality control processing and find homologies to the SILVA 16S rRNA databases (Quast et al., 2012), the metadata file was selected, the project was given a name, fastq sequencing files were selected, Phred score cut-off was increased from 15 (default value) to 20, and prior processing level was selected before submitting.

6) After processing, quality control reports, the number of observed homologies (a.k.a., hits), rarefaction curves, and taxonomic analysis for each library was accessed by following the path download > search website > dataset name.

7) The Analysis Tool option was used to combine data and to choose data sets to assign operational taxonomic units (OTU).

8) To transfer the OTU count data and corresponding taxonomy from MG-RAST, the Analysis page was set to species-level taxonomy, and the tab separated values (TSV) file was downloaded.

9) The TSV file was imported into a spreadsheet, sorted, and unwanted taxa (i.e., eukaryotes, viruses, OTU present in the DNA-free controls) deleted.

10) The process data were arranged in Microbiome Analysis formats (Dhariwal et al., 2017) and exported as a tab-delimited TXT file. Files produced were OTU count data, SILVA taxonomy, and metadata. The data sets used are posted in Supplementary Table S2.

| Bottlenecks | Comment and Resolution |
|---|---|
| Time for next-generation sequencing | Most commercial next-generation sequencing services can require 4 to 6 weeks to sequence samples. As a result, when designing the course, the instructor should consider:<br>• Performing environmental DNA extraction early in the course.<br>• Using the wait-time for the class to perform related experiments. In this course, the students conducted chemical analyses of water samples.<br>• Using real-time technologies such as Oxford Nanopore (Oxford, United Kingdom) MinION sequencing (Brown et al., 2017; Mitsuhashi et al., 2017). |
| Uploading metadata into MG-RAST (Meyer et al., 2008). | The metadata formatting requirements for MG-RAST is precise and somewhat cryptic. As a result, student submissions of the metadata file often failed validation. The following strategies may help overcome the bottleneck:<br>• Download the metadata Excel template provided by MG-RAST<br>• Have students watch the metadata entry video tutorial produced by Argonne National Laboratory (2012).<br>• Have multiple students work on metadata entry. Once a student is successful in having their metadata file validated, share that file with the other students. |
| Time for MG-RAST homology search | Because of the high volume of work on the MG-RAST supercomputer complex (Meyer et al., 2017), it can take a few days from the time sequence data is uploaded into MG-RAST until the operational taxonomic unit (OTU) counts are produced. To reduce the time for data processing:<br>• Only submit sequence data files once. The students can aggregate sequence datasets using identification numbers generated by MG-RAST.<br>• Choose the option of making the sequence data immediately available. The data queue processing algorithm places the highest priority on public data. |
| Data Analysis on MG-RAST | Because of the huge dataset in MG-RAST and its high demand on classroom wireless internet, the students may experience difficulties using the analysis tools. To remedy the bottleneck:<br>• Export the OTU count data at the species level as a tab-delimited file.<br>• Use a spreadsheet to delete undesired data (e.g., eukaryotes).<br>• Use spreadsheet software to format the data file into a format compatible with MicrobiomeAnalyst (Dhariwal et al., 2017; Chong et al., 2020).<br>• Use MicrobiomeAnalyst to perform data normalization and analysis. |

## References


Argonne National Laboratory (2012). The MG-RAST Metadata Spreadsheet http://youtu.be/8YT9tbbHwRA [Accessed January 20, 2020].

Argonne National Laboratory (2017). MG-RAST tutorial: Submitting a dataset http://youtu.be/A06lBNhnupM [Accessed January 20, 2020].

Brown, B.L., Watson, M., Minot, S.S., Rivera, M.C., and Franklin, R.B. (2017). MinION nanopore sequencing of environmental metagenomes: A synthetic approach. *GigaScience* 6**,** 1-10.

Chong, J., Liu, P., Zhou, G., and Xia, J. (2020). Using MicrobiomeAnalyst for comprehensive statistical, functional, and meta-analysis of microbiome data. *Nat. Protoc.* 15**,** 799-821.

Dhariwal, A., Chong, J., Habib, S., King, I.L., Agellon, L.B., and Xia, J. (2017). MicrobiomeAnalyst: a web-based tool for comprehensive statistical, visual and meta-analysis of microbiome data. *Nucleic Acids Res.* 45**,** W180-W188.

Meyer, F., Bagchi, S., Chaterji, S., Gerlach, W., Grama, A., Harrison, T., Paczian, T., Trimble, W.L., and Wilke, A. (2017). MG-RAST version 4: Lessons learned from a decade of low-budget ultra-high-throughput metagenome analysis. *Brief. Bioinformatics* 20**,** 1151-1159.

Meyer, F., Paarmann, D., D'Souza, M., Olson, R., Glass, E.M., and Kubal, M. 2008. The metagenomics RAST server: A public resource for the automatic phylogenetic and functional analysis of metagenomes. *BMC Bioinformatics*, 9:386. doi: 10.1186/1471-2105-9-386.

Mitsuhashi, S., Kryukov, K., Nakagawa, S., Takeuchi, J.S., Shiraishi, Y., Asano, K., and Imanishi, T. 2017. A portable system for rapid bacterial composition analysis using a nanopore-based sequencer and laptop computer. *Sci. Rep.*, 7:5657. doi: 10.1038/s41598-017-05772-5.

Quast, C., Pruesse, E., Yilmaz, P., Gerken, J., Schweer, T., Yarza, P., Peplies, J., and Glöckner, F.O. (2012). The SILVA ribosomal RNA gene database project: Improved data processing and web-based tools. *Nucleic Acids Res.* 41**,** D590-596.
