# Supplementary material for: Students in a Course-Based Undergraduate Research Experience Course Discovered Dramatic Changes in the Bacterial Community Composition Between Summer and Winter Lake Samples
Source: Front Microbiol. 2021 Feb 18;12:579325. doi: 10.3389/fmicb.2021.579325 (PMC7929996; doi:10.3389/fmicb.2021.579325)
Supplement: Supplementary Table 3 — Results of anonymous end-of-course student questionnaire. [file Table_3.pdf]

Table S3: Results of anonymous end of course student questionnaire.

2019 Student Responses:

1) What aspects of the course did you like?
   - Relaxing learning environment to ask questions and learn at an individualized pace. New research techniques. Field experiments!
   - The field study & teamwork
   - On hands work & doing experiments ourselves. Field trip. The professor is cool. Helpful lab report outlines.
   - Collecting samples and working to find out new information.
   - I learned hour to drill holes in ice so I loved the field collection aspect of the class.
   - I loved how we worked together for each experiment. Working together helped making the experiments more enjoyable and less stressful.
   - That is was exploratory. Hands on. We get to work independently in groups. Was able to learn w/o stress.

2) What changes can be made to improve the course?
   - Get a final exam period. Group quiz!
   - More field experiences
   - Organized lectures
   - Things seemed to take longer than expected so we ended up not getting through everything we planned to.
   - Maybe more organization or pick (as a class) what we research
   - Better organization of what needs to be done. Better precautions to not contaminate experiments.
   - The timing. I think it would be more efficient w/ a better time slot.

2020 Student Responses:

1) What aspects of the course did you like?
   - it was very knowledgable course and i learned a lot from it! Dr. Baker is a brilliant teacher who helps his students excel in the class.
   - I liked how it combined wet lab and computer analysis which gave students a chance to do research techniques.
   - it was fun
   - the lab was more of a large group working all together because everyone was assisting everyone else
   - having hands-on and conducting the study on our own
   - I enjoyed performing an experiment without an expected outcome. This reflects legitimate research not cookie cutter school labs people normally do.
   - The hands on appraoch of this labratory and the ablity to learning from your mistakes in a laboratory setting.
   - that we had freedom and it was an upper level lab with the ability to choose our own

partners and we are able to follow protocols and edit mistakes and stuff

- One of my favorite aspects of this course was isolating DNA. I really enjoyed learning about how to isolate and purify DNA prior to amplification and sequencing. I will believe that learning this will greatly benefit me in future courses. Even though we had to perform this protocol twice, we managed to acquire high DNA yields the second time, and I felt much more confident about my ability to perform this protocol without the proctor/teacher's assistance.
- i liked the i knew about a new material that i never thought it existed and i can go deeply in things i never had an idea about
- The content of the class
- That we were able to revise our lap reports.
- that the main class was based solely on research and working actively
- I liked that it was a fast-growing science field and that we got to explore a research article of our liking and present it at the end of the course. I enjoyed learning new lab techniques and spending time perfecting them at the beginning of the course - that was very helpful for the remainder of the course.

2) What changes can be made to improve the course?
- more online resources to help students understand certain procedures and methods.
- Perhaps homework (not assessments) that would be due the next lab session so students can make sure they know what is going on in lab idea wise.
- worth more credits
- have a better explanation of the material before starting
- This could be improved by maybe improving preparedness or having a better TA.
- The meeting times or credits need to be adjusted and more laboratory equipment is required.
- nothing, it was awesome!
- The course went smoothly overall; however, my biggest suggestion is to make this course meet once a week from 1-5 rather than the current meeting times. As a commuter, this was really difficult for me to come twice a week especially since I had no other classes on Thursdays. Many class sessions were just a form of recitation explaining what we will be doing in the upcoming days/weeks. Thus, I believe that switching to 4 hour s once a week
- the lab reports are a little too heavy for what we have covered so far i think 2 lab reports are more than enough
- One of the two class periods should be a longer style recitation
- More time for conducting our experiments.
- being able to submit actual papers to scientific journals
- I think having more of a set plan of procedures would be helpful. But it is very hard to do so since we had to repeat some experiments. Other than that I think the course went well.
