# Supplementary material for: Students in a Course-Based Undergraduate Research Experience Course Discovered Dramatic Changes in the Bacterial Community Composition Between Summer and Winter Lake Samples
Source: Front Microbiol. 2021 Feb 18;12:579325. doi: 10.3389/fmicb.2021.579325 (PMC7929996; doi:10.3389/fmicb.2021.579325)
Supplement: Supplementary Table 4 — Overcoming bottlenecks in student workflow. [file Table_4.pdf]

Table S4: Overcoming bottlenecks in student workflow

Bottlenecks	Comment and Resolution
Time for next-generation sequencing	Most commercial next-generation sequencing services can require 4 to 6 weeks to sequence samples. As a result, when designing the course, the instructor should consider:<br>• Performing environmental DNA extraction early in the course.<br>• Using the wait-time for the class to perform related experiments. In this course, the students conducted chemical analyses of water samples.<br>• Using real-time technologies such as Oxford Nanopore (Oxford, United Kingdom) MinION sequencing (Brown et al., 2017; Mitsuhashi et al., 2017).
Uploading metadata into MG-RAST (Meyer et al., 2008).	The metadata formatting requirements for MG-RAST is precise and somewhat cryptic. As a result, student submissions of the metadata file often failed validation. The following strategies may help overcome the bottleneck:<br>• Download the metadata Excel template provided by MG-RAST<br>• Have students watch the metadata entry video tutorial produced by Argonne National Laboratory (2012).<br>• Have multiple students work on metadata entry. Once a student is successful in having their metadata file validated, share that file with the other students.
Time for MG-RAST homology search	Because of the high volume of work on the MG-RAST supercomputer complex (Meyer et al., 2017), it can take a few days from the time sequence data is uploaded into MG-RAST until the operational taxonomic unit (OTU) counts are produced. To reduce the time for data processing:<br>• Only submit sequence data files once. The students can aggregate sequence datasets using identification numbers generated by MG-RAST.<br>• Choose the option of making the sequence data immediately available. The data queue processing algorithm places the highest priority on public data.
Data Analysis on MG-RAST	Because of the huge dataset in MG-RAST and its high demand on classroom wireless internet, the students may experience difficulties using the analysis tools. To remedy the bottleneck:<br>• Export the OTU count data at the species level as a tab-delimited file.<br>• Use a spreadsheet to delete undesired data (e.g., eukaryotes).<br>• Use spreadsheet software to format the data file into a format compatible with MicrobiomeAnalyst (Dhariwal et al., 2017; Chong et al., 2020).<br>• Use MicrobiomeAnalyst to perform data normalization and analysis.

References

Argonne National Laboratory (2012). The MG-RAST Metadata Spreadsheet http://youtu.be/8YT9tbbHwRA [Accessed January 20, 2020].

Argonne National Laboratory (2017). MG-RAST tutorial: Submitting a dataset http://youtu.be/A06lBNhnupM [Accessed January 20, 2020].

Brown, B.L., Watson, M., Minot, S.S., Rivera, M.C., and Franklin, R.B. (2017). MinION nanopore sequencing of environmental metagenomes: A synthetic approach. *GigaScience* 6**,** 1-10.

Chong, J., Liu, P., Zhou, G., and Xia, J. (2020). Using MicrobiomeAnalyst for comprehensive statistical, functional, and meta-analysis of microbiome data. *Nat. Protoc.* 15**,** 799-821.

Dhariwal, A., Chong, J., Habib, S., King, I.L., Agellon, L.B., and Xia, J. (2017). MicrobiomeAnalyst: a web-based tool for comprehensive statistical, visual and meta-analysis of microbiome data. *Nucleic Acids Res.* 45**,** W180-W188.

Meyer, F., Bagchi, S., Chaterji, S., Gerlach, W., Grama, A., Harrison, T., Paczian, T., Trimble, W.L., and Wilke, A. (2017). MG-RAST version 4: Lessons learned from a decade of low-budget ultra-high-throughput metagenome analysis. *Brief. Bioinformatics* 20**,** 1151-1159.

Meyer, F., Paarmann, D., D'Souza, M., Olson, R., Glass, E.M., and Kubal, M. 2008. The metagenomics RAST server: A public resource for the automatic phylogenetic and functional analysis of metagenomes. *BMC Bioinformatics*, 9:386. doi: 10.1186/1471-2105-9-386.

Mitsuhashi, S., Kryukov, K., Nakagawa, S., Takeuchi, J.S., Shiraishi, Y., Asano, K., and Imanishi, T. 2017. A portable system for rapid bacterial composition analysis using a nanopore-based sequencer and laptop computer. *Sci. Rep.*, 7:5657. doi: 10.1038/s41598-017-05772-5.

Quast, C., Pruesse, E., Yilmaz, P., Gerken, J., Schweer, T., Yarza, P., Peplies, J., and Glöckner, F.O. (2012). The SILVA ribosomal RNA gene database project: Improved data processing and web-based tools. *Nucleic Acids Res.* 41**,** D590-596.
